# The effect of alcohol marketing on people with, or at risk of, an alcohol problem: A rapid review

**DOI:** 10.1093/eurpub/ckac131.327

**Published:** 2022-10-25

**Authors:** T Langley, J Leonardi-Bee, A Barker, O Brown, R Murray

**Affiliations:** School of Medicine, University of Nottingham, Nottingham, UK

## Abstract

**Background:**

Little is known about the impact of alcohol marketing on people with, or at risk of, an alcohol problem. A rapid review of primary studies was conducted with the aim of exploring the effect of alcohol marketing in this population.

**Methods:**

People with, or at risk of an alcohol problem were defined as people with an alcohol use disorder, in recovery from an alcohol use disorder, and hazardous and harmful drinkers. Searches for relevant literature were conducted through Medline, EMBASE and PsychINFO; reference list scanning and citation tracking of included studies; and grey literature searching of relevant websites. A narrative synthesis of included studies was undertaken.

**Results:**

The review included 11 studies, which focused on participants recovering from an alcohol use disorder (AUD, 6 studies) and those with hazardous or harmful consumption levels of alcohol (5 studies). 7 studies were quantitative and 4 were qualitative. The effect of alcohol advertising on alcohol use was only assessed in one small experimental study of young adult heavy drinkers, which found no apparent effect. Studies looking at other outcomes suggested that a significant proportion of people with or at risk of alcohol problems notice alcohol advertisements and can find them appealing, and that advertisements may have an effect on positive alcohol-related emotions and cognitions. Among people in recovery from an alcohol use disorder, findings suggested that there could be an effect on craving, and that alcohol marketing may be perceived to trigger a desire to drink.

**Conclusions:**

Several studies report effects of alcohol marketing which may translate into effects on consumption. There is also evidence that alcohol marketing is perceived to act as a trigger by people in recovery from alcohol problems. Further longitudinal and experimental research is needed to determine whether alcohol marketing has a causal effect on alcohol use in this population.

**Key messages:**

• The findings of the studies included in the review suggest that an effect of alcohol marketing in people with, or at risk of, an alcohol problem is likely.

• The impact of alcohol marketing on people with or at risk of an alcohol problem should be a concern for marketing regulators and a focus for future research.

